# Severe re-expansion pulmonary edema despite incomplete re-expansion of the lung: a case report

**DOI:** 10.1186/s13256-021-03112-w

**Published:** 2021-10-16

**Authors:** Alicja Zabielna

**Affiliations:** Addenbrookes Hospital, Hills Road, Cambridge, CB2 0QQ UK

**Keywords:** Re-expansion, Pulmonary edema, Pneumothorax

## Abstract

**Background:**

Re-expansion pulmonary edema is a rare but potentially fatal (mortality up to 20%) complication of the rapid removal of air, fluid, or other space-occupying lesion from the pleural cavity. This case report highlights the fact that this complication is much more likely to occur when treating large, chronic pneumothoraces, and can occur even if the lung fails to fully re-expand.

**Case presentation:**

A 49-year-old white British man presented to the emergency department with sudden onset of shortness of breath 5 days prior to admission. A left-sided pneumothorax was suspected on clinical examination, and chest X-ray confirmed a large, left-sided pneumothorax. A 12 French gauge chest drain was inserted and connected to an underwater seal. Shortly after insertion of the drain, the patient’s condition deteriorated rapidly with tachypnea and severe hypoxemia. A diagnosis of re-expansion pulmonary edema was made, and the patient was treated with high-flow oxygen and continuous positive airways pressure.

**Conclusions:**

This case report serves as a reminder of the morbidity and potential mortality associated with a commonly performed medical procedure, and reveals a lack of clear and precise guidance on the management of large, chronic (> 72 hours) pneumothoraces in the current British Thoracic Society pleural disease guidelines.

## Summary

**Fig. 1:**
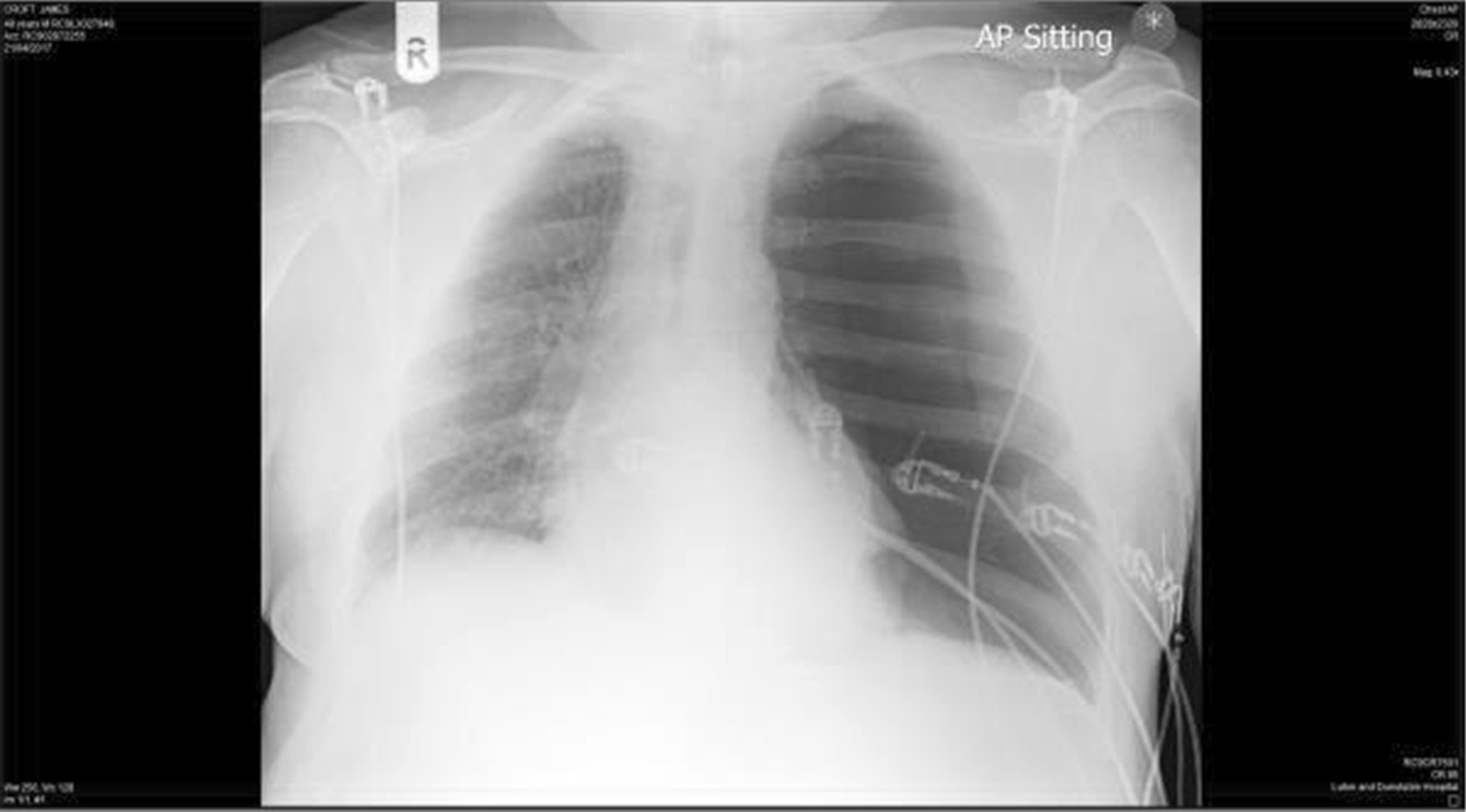
CXR showing large, left-sided pneumothorax

A 49-year-old white British man presented to the emergency department with sudden onset of shortness of breath 5 days prior to admission. A left-sided pneumothorax was suspected on clinical examination, and chest X-ray (CXR) confirmed a large, left-sided pneumothorax (Fig. 1). A 12 French gauge chest drain was inserted and connected to an underwater seal. Shortly after insertion of the drain, the patient’s condition deteriorated rapidly with tachypnea and severe hypoxemia. A diagnosis of re-expansion pulmonary edema was made, and the patient was treated with high-flow oxygen and continuous positive airways pressure (CPAP).

This case serves as a reminder of the morbidity and potential mortality associated with a commonly performed medical procedure, and reveals a lack of clear and precise guidance on the management of large, chronic (> 72 hours) pneumothoraces in the current British Thoracic Society (BTS) pleural disease guidelines [1, 2].

## Background

Spontaneous pneumothorax is a relatively common and important health problem, nationally and globally, with around 8000 admissions a year in the UK [3], many of which are treated by thoracocentesis or tube thoracostomy. Re-expansion pulmonary edema (RPO) is a rare (< 1%, but up to 14% in some case series [4]) but potentially fatal complication of the rapid removal of air, fluid, or other mass from the pleural cavity, with a reported mortality rate as high as 20% [5].

It is recognized that the risk of developing RPO is increased in chronic (> 72 hours) pneumothoraces [5–8], and that the risk is further increased the larger the pneumothorax [7–13], with a rate of up to 44% in tension pneumothoraces. A high index of suspicion is therefore necessary when faced with a large, chronic pneumothorax.

While current (2010) BTS guidelines recognize RPO as a potential complication of pleural drainage, they do not *emphasize* the fact that the risk of this complication is significantly increased in large and chronic pneumothoraces, and thereby do not raise the readers’ index of suspicion in such cases.

The guidelines recommend stopping pleural aspiration when no more fluid or air can be aspirated, the patient develops symptoms of cough or chest discomfort, or 1.5 L has been withdrawn. In the case of pleural effusion, it is relatively easy to observe and measure the volume of fluid withdrawn. The guidelines do not, however, suggest how the volume of air aspirated from a pneumothorax through an underwater seal should be measured, and there are no suggestions as to how a large pneumothorax can be drained slowly.

It has been shown in animal models that the risk of developing RPO can be minimized, and larger volumes can be drained, if the pleural pressure is kept above −20 cm H_2_O, but this obviously requires the use of pleural manometry, which is not currently routinely practiced in the UK.

## Case presentation

**Fig. 2:**
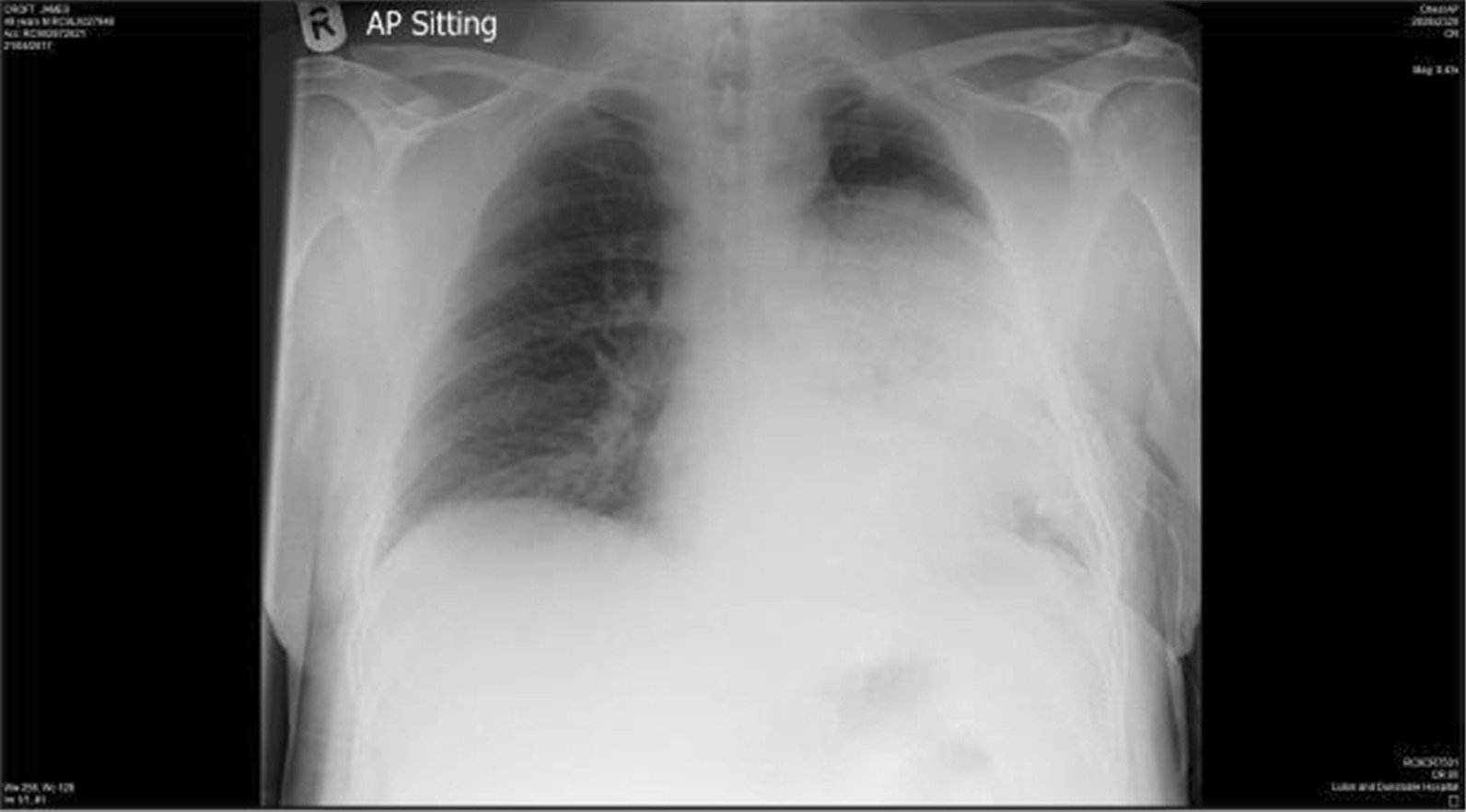
Repeat CXR post chest drain insertion showing incomplete re-expansion of the lung with dense alveolar shadowing

A 49-year-old white British male smoker presented to the emergency department with a history of sudden onset of shortness of breath associated with a cough productive of brown sputum 5 days prior to admission. He had been treated with antibiotics by his general practitioner without any improvement. On admission, his respiratory rate was 38 breaths per minute, with oxygen saturation of 91% on room air. Initial arterial blood gas (ABG) revealed a pO_2_ of 10.2 kPa (normal > 10.6 kPa on room air). Clinically, he was noted to have reduced air entry on the left, and CXR showed a complete left-sided pneumothorax (Fig. 1). A 12 Fr chest drain was inserted and allowed to drain freely through an underwater seal. Shortly after insertion of the drain, the patient appeared to become unwell, with worsening breathlessness and signs of hypoxia. Oxygen saturation dropped to 83%, and a repeat ABG showed a pO_2_ of 6.4 kPa while breathing 4 L per minute of oxygen via nasal cannula. A repeat CXR showed that the left lung had re-expanded by about 70%, with dense alveolar shadowing (Fig. 2), and the diagnosis of re-expansion pulmonary edema was made.

## Treatment

The patient was initially treated with high-flow oxygen, intravenous furosemide, and hydrocortisone. He was then transferred to the high dependency unit (HDU) and, as he had a functional chest drain *in situ*, was started on continuous positive airways pressure (CPAP). In spite of CPAP and increasing the FiO to 95%, he did not show any improvement in the first 6 hours of treatment. After 6 hours, he began to make slow but steady improvement. He remained on CPAP on HDU for the next 6 days, but despite a functioning chest drain, the lung failed to expand fully, and he was subsequently transferred to a thoracic surgical unit for consideration of surgical intervention.

## Outcome and follow-up

After transferring to the thoracic surgical unit, he had a repeat CXR that showed that the lung was expanded fully, requiring no further treatment, and the chest drain was removed subsequently. He was discharged without any surgical intervention.

## Discussion and conclusions

Re-expansion pulmonary edema has been recognized as a potential complication of thoracocentesis for over 150 years. It was first described in relation to *pleural effusion* thoracocentesis by Pinault in 1853 [14], but it was not until 1958 that Carlson *et al.* reported it in the context of pneumothorax [15]. In 1988, a case review by Mahfood and associates [5] found 11 fatalities in 53 cases reviewed, giving a mortality rate of 20%, and this figure is quoted in much of the literature surrounding RPO, including the 2010 BTS guidelines. While the incidence of RPO is thought generally to be less than 1%, in some case series it is much higher than this [7–11], especially in large, chronic pneumothoraces, and this group should be considered as high risk for the development of RPO.

While the treatment of RPO is mainly supportive (and is in most cases effective), considering its potential high mortality (up to 20%), the main aim should be in preventing this complication in clinical practice. No randomized clinical trials comparing different methods of draining pneumothoraces are published, but many articles make the assumption that the rapidity of drainage may be the cause for development of RPO and they suggest a slow, controlled re-expansion especially in the case of large, chronic pneumothoraces. Current BTS guidelines suggest that the process of re-expanding the lung should be stopped when no more fluid or air can be aspirated, the patient develops symptoms of cough or chest discomfort, or 1.5 L has been withdrawn [1]. Obviously in the case of pleural effusion, the amount of fluid can be easily measured, but this is not the case during drainage of a pneumothorax through a thoracostomy tube draining freely through an underwater seal.

Feller-Kopman argued in 2012 [16] that pleural manometry should be used routinely during thoracocentesis, stating that it adds useful clinical information that would impact management, is easy to perform, has few risks to the patient, and does not significantly add to the costs. He suggests that pleural manometry provides information regarding the ability of the lung to re-expand during thoracocentesis (the lung in our case failed to re-expand fully). He further indicates that it could be used to guide large-volume thoracocentesis in selected cases.

Chen *et al.* [17] suggested the use of pigtail catheters attached to three-way taps and intermittent drainage in the case of prolonged, massive pneumothorax.

However, if the pneumothorax is drained, in the case of chronic (> 72 hours), large pneumothorax, it is crucial that the operator is aware of the chronicity of the pneumothorax, and maintains a high index of suspicion for the development of RPO.

## Learning points

1. Spontaneous pneumothorax requiring thoracocentesis or tube thoracostomy is common.
2. RPO is a rare but *potentially fatal* iatrogenic complication of thoracocentesis or tube thoracostomy, and most cases occur after treatment of pneumothoraces.
3. It is more likely to occur after the treatment of large, chronic (> 72 hours) pneumothoraces, and a high index of suspicion should be maintained in such cases.
4. It can occur even if the lung does not re-expand fully.
5. Current BTS guidelines do not clearly emphasize the increased risk in large, chronic pneumothoraces, and lack clarity on how such cases should be managed.

## Data Availability

Not applicable.
